# Application of High-Performance Liquid Chromatography for Simultaneous Determination of Tenofovir and Creatinine in Human Urine and Plasma Samples

**DOI:** 10.3390/ph13110367

**Published:** 2020-11-05

**Authors:** Patrycja Olejarz, Grażyna Chwatko, Paweł Kubalczyk, Krystian Purgat, Rafał Głowacki, Kamila Borowczyk

**Affiliations:** Department of Environmental Chemistry, Faculty of Chemistry, University of Lodz, 163 Pomorska Str., 90-236 Łódź, Poland; patrycja.olejarz@o2.pl (P.O.); grazyna.chwatko@chemia.uni.lodz.pl (G.C.); pawel.kubalczyk@chemia.uni.lodz.pl (P.K.); krystianpurgat@gmail.com (K.P.); rafal.glowacki@chemia.uni.lodz.pl (R.G.)

**Keywords:** tenofovir, creatinine, HPLC-UV, hepatitis B virus, human immunodeficiency virus

## Abstract

Tenofovir disoproxil fumarate is widely used in the therapy of human immunodeficiency virus and hepatitis B virus; however, a high concentration of the prodrug effects kidney function damage. To control the effectiveness of kidney functions in treated patients, the level of creatinine in the body must be controlled. This work describes a simple, fast, and “plastic-waste” reducing method for the simultaneous determination of tenofovir and creatinine in human urine and plasma. In both assays, only 50 µL of body fluid was required. The tests were carried out by reversed phase high-performance liquid chromatography with UV detection. In urine samples, the limits of detection for tenofovir and creatinine were 4 µg mL^−1^ and 0.03 µmol mL^−1^, respectively. In plasma samples, the limits of detection were 0.15 µg mL^−1^ for tenofovir and 0.0003 µmol mL^−1^ for creatinine. The method was applied for the determination of tenofovir and creatinine in human urine and plasma samples. The biggest advantage of the elaborated method is the possibility to determine tenofovir and creatinine in one analytical run in both urine and plasma sample collected from HIV and HBV patients. The possibility to reduce the level of laboratory waste in a sample preparation protocol is in the mainstream of a new trend of analytical chemistry which is based on green chemistry.

## 1. Introduction

The report presented by the Joint United Nations Program on HIV/AIDS (UNAIDS) in 2018 shows that 36.9 million people globally are living with human immunodeficiency virus (HIV) [1]. The World Health Organization (WHO) found that, worldwide, 257.0 million persons were living infected with hepatitis B virus (HBV) in 2016, and 1.3 million deaths were caused by the virus in 2015 alone. Both viruses are major public health problems that require an urgent response [2].

HBV infection is caused by the virus belonging to the hepadnavirus family, one of the smallest viruses known to infect humans. The enveloped DNA virus infects liver cells, causing hepatocellular necrosis and inflammation [2,3]. HIV infects immune system cells and is able to destroy or impair their functions [1].

To stop the worldwide transmission of HBV and HIV, the WHO recommends the use of antiretroviral (ARV) treatment for infected people and the application of ARV drugs to prevent the mother-to-child transmission of HIV [2,3,4,5]. Currently, to treat patients with chronic hepatitis B (CHB), HBV or HIV, or people living with HBV-HIV coinfection, seven antiviral agents have been recommended. One of those is tenofovir disoproxil fumarate, which is an orally available bioactive prodrug of tenofovir (TFV) [6]. TFV is a nucleotide analog of reverse transcriptase, which is very effective in therapy against retroviruses and hepadnaviruses [7]. It was approved by the US Food and Drug Administration for the treatment of infections: HIV in 2001 and CHB in 2008. Currently, TFV is recommended as one of the first drugs in monotherapy of CHB [8]. Statistical data presented by the WHO have shown a crucial delay in the progression of cirrhosis, reduction in the incidence of CHB and improvement of long-term survival in people living with HBV treated with TFV [3]. Generally, TFV is well tolerated. However, some evidence of a decrease in bone mineral density, changes in kidney functions and in the rate of tubular dysfunction after treatment with TFV has been reported [9,10]. TFV treatment has been confirmed to be associated with a higher risk of nephrotoxicity in clinical cohorts [11,12]. Several studies have shown a raised prevalence of proximal renal tubular dysfunction in TFV-treated patients, attributed to increased intracellular TFV concentrations and direct mitochondrial toxicity in the proximal tubule cells [13,14].

The first case of nephrotoxicity induced by TFV in a patient with HIV was reported in 2002 [15]. Since that time, severe or symptomatic nephrotoxicity has also been reported in CHB patients treated with TFV [16,17,18,19,20]. That was proven that even short-term therapy with this drug results in severe renal dysfunction [20]. Due to the nephrotoxicity of TFV, every initiation of the drug treatment must be preceded by renal function control. Additionally, more frequent monitoring of TFV level and kidney functions in TFV-treated patients at higher risk of renal dysfunction is recommended by the WHO [3].

The guideline on TFV monitoring in HBV patients published by the European Liver Research Association recommends an estimation of glomerular filtration rate (eGFR) before starting the TFV therapy. The eGFR control is based on comparison of levels of creatinine (Crn) in plasma and urine. Monitoring of eGFR in all patients treated with TFV every 1–3 months during the first year of treatment and then every 3–6 months is recommended by the WHO [3,21].

The renal clearance of Crn is one of the most used and commonly accepted tests of renal function [22]. Since TFV is mainly excreted by tubular secretion and it is active on the tubular cells, its urinary concentration could be a useful marker of TFV-associated tubular toxicity and a reasonable candidate for clinical use [21]. Additional tests for Crn concentration in urine and plasma are helpful to control the bodily absorption, distribution, metabolism and TFV excretion.

Since HIV and HBV infections are possible through blood samples, the use of urine in place of plasma for body TFV monitoring seems to be safer, more significant and deeply required [2,3,22]. Utilization of urine samples significantly reduces the risk of random infections associated with the transport, storage or disposal of infected samples compared to plasma. Moreover, for studying the effect of antiviral therapy on kidney functions, it is necessary to determine the Crn content in urine.

The lack of information about analytical protocols dedicated for simultaneous quantitation of TFV and Crn indicates the need to develop an essential assay for the measurement of side-effects/interactions and optimization of treatment protocols of HIV and HBV/CHB patients. In this paper, we present a new analytical tool based on reversed-phase high-performance liquid chromatography (RP-HPLC) with UV detection for the direct and simultaneous determination of Crn and TFV in urine samples collected from CHB patient treated with TFV and plasma samples spiked with this drug.

## 2. Results

### 2.1. Chromatpgraphy

**Figure 1:**
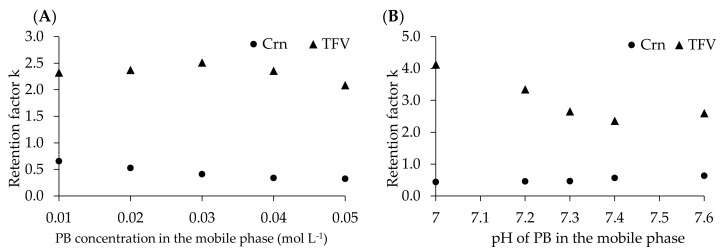
The influence of phosphate buffer (PB) concentration (**A**) and pH (**B**) in the mobile phase on the retention factors of creatinine (Crn) and tenofovir (TFV). Chromatographic conditions in Section 4.5.

RP-HPLC is one of the most common analytical techniques dedicated to biological sample analysis. The choice of chromatographic conditions directly affects the quality of analyte separation. The amounts of organic and inorganic solvents in a mobile phase are crucial from the chromatographic, economic and environmental points of view. To reduce the amount of toxic waste in this analysis, we decided to use low-concentration phosphate buffer (PB) and a small amount of acetonitrile (MeCN). The mobile-phase pH can be a powerful tool to control retention and selectivity. Hence, we studied concentrations of PB in the range from 0.01 to 0.05 mol L^−1^ and its pH in the range from 7.0 to 7.6 (Figure 1). The results were obtained using a urine sample spiked with Crn and TFV. The analysis was performed at 25 °C with a mobile phase flow rate equal to 1 mL min^−1^.

**Figure 2:**
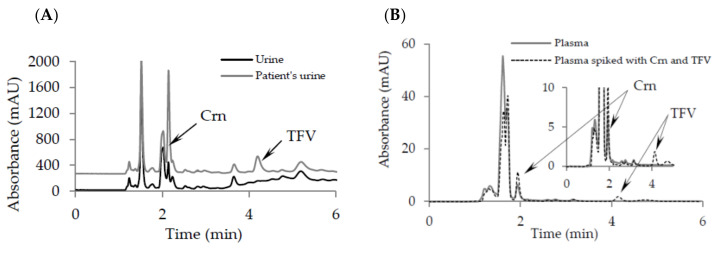
Representative chromatograms of: (**A**) urine, patient’s urine and patient’s urine spiked with TFV; (**B**) plasma and plasma spiked with Crn and TFV. Chromatographic conditions in Section 4.5.

In the case of chromatographic methods based on UV detection, utilization of the most proper wavelengths is crucial for detection of the analyte and the method sensitivity. For this reason, we considered application of two different wavelengths. Detection was carried out by using 234 nm from 0 to 3 min for Crn monitoring and 260 nm from 3 to 8 min for TFV detection. Representative chromatograms of urine and plasma samples have been presented on Figure 2.

### 2.2. Method Validation

#### 2.2.1. Method Calibration

For method calibration, 50 µL of urine samples were placed in glass vials and spiked with 10 µL of growing amounts of working standard solution containing the analytes at seven levels of concentration. The calibration ranges were 10.0–300.0 µg mL^−1^ urine and 0.1–30 µmol mL^−1^ urine for TFV and Crn, respectively. Plasma samples (50 µL) were spiked with the increasing amounts of working solutions of TFV and Crn to provide the final concentration of TFV from 0.5 to 5 µg mL^−1^ plasma and for Crn from 0.001 to 0.04 µmol mL^−1^ plasma. Then, the samples were processed according to the procedures described in Section 4.4. The calibration solutions were prepared in triplicate. The calibration curves were obtained by plotting the peak areas against the analyte concentrations. Regression equations and correlation coefficients have been presented in Table 1.

#### 2.2.2. LOD and LOQ

The limit of detection (LOD) and limit of quantification (LOQ) were defined as the concentrations with a signal-to-noise (S/N) ratio of 3 and 10, respectively [23]. Peaks of the analytes were identified by comparison of spectrum and retention time with parameters obtained for authentic standards. In the method dedicated to urine, LODs were 4 µg mL^−1^ and 0.03 µmol mL^−1^, while LOQs were 8 µg mL^−1^ and 0.1 µmol mL^−1^ for TFV and Crn, respectively. In the method dedicated to the determination of TFV and Crn in plasma, LODs and LOQs were 0.15 and 0.2 µg mL^−1^ for TFV and 0.0003 and 0.001 µmol mL^−1^ for Crn, respectively.

#### 2.2.3. Precision and Accuracy

Precision and accuracy were calculated using the results of the analysis of urine and plasma samples spiked with known amounts of these analytes, analyzed in triplicate. The procedure followed the guidelines for biological sample analysis [23,24]. Precision was expressed in terms of relative standard deviation (RSD), whereas accuracy was considered as the percentage of analyte recovery calculated by expressing the mean measured amount as a percentage of the added amount. The estimated validation parameters for analytes were satisfying. The detailed data are presented in Table 2.

### 2.3. Stability Study

**Figure 3:**
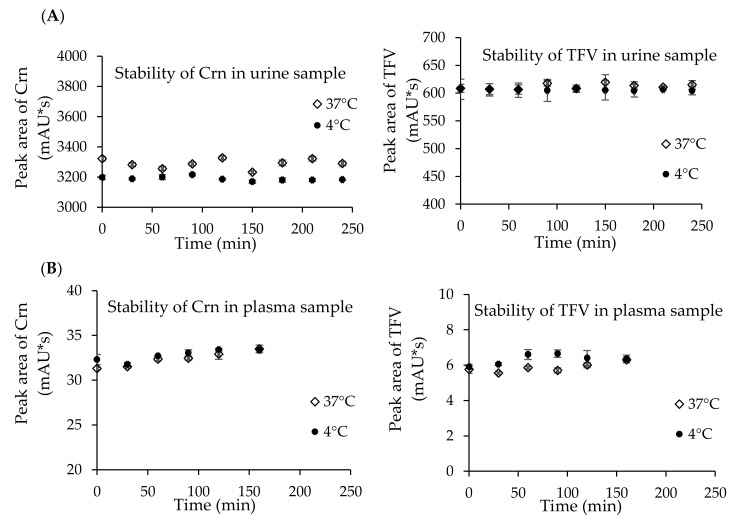
Stability of Crn and TFV in urine (**A**) and plasma (**B**) samples kept at 4 °C and 37 °C; *n* = 3 for each time point.

To confirm the usefulness of the elaborated method, the analyte stability studies were also performed. Urine and plasma samples were spiked with known amounts of analytes and prepared according to protocols described in Section 4.4. Samples were kept at 4 °C—the temperature used during sample storing—and at 37 °C—the temperature close to the human body temperature. Samples were analyzed in 30 min intervals over 3 h for plasma and 4 h for urine. Urine and plasma samples dedicated for stability studies were prepared in triplicate. The obtained data are presented in Figure 3.

### 2.4. Urinary Excretion of TFV

**Figure 4:**
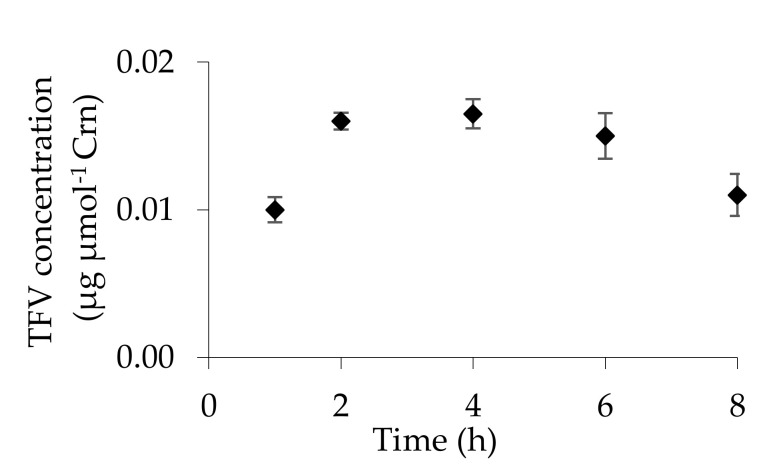
Urinary excretion of TFV after drug intake in dose 123 mg. TFV concentration normalized against Crn; *n* = 3 for each time point.

The method was applied to control the urinary excretion of TFV in one CHB patient treated with TFV in form of Viread 123 mg film-coated tablets. Urine samples were collected after one, two, four, six and eight hours after taking the pharmaceutical dose, prepared in triplicate and analyzed. The concentration of TFV in each sample was simultaneously normalized to Crn. The obtained results are presented in Figure 4.

### 2.5. Carry-Over Assay

To confirm the possibility of the application of reusable/washed HPLC glass vials, the carry-over assay was performed. Carry-over was assessed by injecting two blank proxy matrix placed in glass vials used previously for storage the high concentrated solutions of standards (300 µg mL^−1^ for TFV and 30 µmol mL^−1^ for Crn). No peaks at the retention times of the analytes were found. Since Crn is an endogenous compound present in human urine, in this experiment we used a proxy matrix as the blank samples.

## 3. Discussion

Due to high individual variability of pharmacokinetic in different patients treated with the same dose of TFV monitoring of TFV level in HIV and HBV patient’s body fluids is recommended by the WHO [3,25]. The dissimilarity is related to the quality and speed of metabolism and interactions between drugs. TFV concentration in plasma affects its action in the human body. Too low a level of the drug may lead to immunization of the virus, while too high concentration significantly increases its renal toxicity [9,10]. Monitoring of TFV in HIV patients is required in controlling the therapeutic dose and in relation to the control of normal renal functions [26,27]. In patients with impaired renal function, it is necessary to adjust the dose of the drug, due to its potential toxicity. The control is performed by Crn clearance study [28,29].

Testing of TFV level in human peripheral blood might be painful for patients and carries the risk of additional HIV/HBV infections. Methods created for TFV determination in non-infected matrices, such as urine [28,30] or hair [31,32], do not provide the possibility for simultaneous monitoring concentration of Crn. Previously published reports mainly described methods for the determination of TFV in plasma [25,26,29,33,34,35,36,37,38,39,40], which determines a higher risk of infection. These assays were based on a solid phase extraction [35,41,42,43] or derivatization [37].

Additional steps in analytical protocols usually increase the number of used tubes, tips, polypropylene vials or columns for solid phase extraction. We proposed the preparation of urine samples directly in HPLC glass vials. To confirm if this can produce a carry-over effect, we performed an additional test. Using the same glass vial for the analysis of standard solutions and next for proxy matrix analysis, we did not observe peaks at the retention times of the analytes in the blank samples. Additionally, precision and accuracy presented in Table 2 show the low possibility of a carry-over effect for the method. We confirmed that even at the highest concentration, there were no adsorption issues related to glass and it was possible to evaluate the LOQ level. This indicates that the goals of the reduction in plastic waste and the possibility to reuse the glass vials have been obtained. In the literature, only two methods are known to be useful for the determination of TFV in urine samples [28,30]; however, none of these allow for the determining of Crn. For decreasing the hazardous properties of the matrix collected from HIV or HBV/CHB patients, analysis of urine samples is more desirable. The acquisition and preparation of urine are safer and do not pose a threat of HIV infection.

Previously described assays dedicated for TFV determination are based on RP-HPLC, usually coupled with mass spectrometry [25,29,32,37,39,40]. Methods requiring commonly available detectors such as spectrophotometric [35,36] or spectrofluorimetric [33,34] detectors are used rarely.

To obtain the most acceptable results of chromatographic separation, various analytical columns, including Aeris WIDEPORE XB-C18 (150 × 4.6 mm, 5 µm), Poroshell (75 × 4.6 mm, 2.7 µm), Kinetex HILIC (100 × 4.6 mm, 2.6 µm) and Zorbax SB C-18 (150 × 4.6 mm, 5 µm) were tested. The choice was based on information that Aeris WIDEPORE column enables the analysis of plasma samples without previous deproteinization [44]. HILIC is one of the most commonly applied methods to solve retention problems of highly polar analytes [45]. Due to the high polarity of the tested compounds, we evaluated the use of a HILIC column. A typical mobile phase for HILIC chromatography includes water-miscible polar organic solvents such as MeCN with a small amount of water [45]. Alcohols can also be adopted, although a higher concentration is needed to achieve the same degree of retention of the analyte relative to an aprotic solvent–water combination [46]. HILIC separations are performed either in isocratic mode with a high percentage of organic solvent or with gradients starting with a high percentage of organic solvent and ending with a high proportion of aqueous solvent [47]. To obtain the best resolution of the analytes for each column, several mobile phase variations were investigated, and initially the effect of organic modifiers such as methanol and MeCN was evaluated. Our aim was to obtain sharp peaks within an acceptable analysis time and to minimize the amount of organic modifier in the mobile phase. The best separation with the symmetrical peak shapes was performed using the Zorbax SB C-18 (150 × 4.6 mm, 5 µm) column. To provide optimal separation between eluted compounds, MeCN was selected as an organic modifier and the gradient mode of separation was performed.

To the best of our knowledge, the HPLC-UV method described herein is the first that makes it possible to determine both Crn and TFV in urine or plasma samples in one analytical run. The total time for urine sample preparation and analysis is less than 10 min, and is shorter than in previous assays [25,30,35,36]. The proposed assay provides quick determination of TFV and simultaneous normalization against Crn concentration. This significantly shortens the entire time of the analytical procedure. The proposed analytical conditions allow good separation of analytes from the matrix components. For Crn, the retention time was 2.3 min, and for TFV, it was 4.5 min. Detection was carried out by using two different wavelengths in one analytical run. The gradient of wavelengths improved specificity and sensitivity of the method.

The proposed analytical procedure has been fully validated. The process was based on recommendations of the EMA Guideline on Bioanalytical Method Validation (2015) [23] and the FDA Guidance for Industry Bioanalytical Method Validation (2018) [24]. The obtained data meet the validation requirements (Table 1 and Table 2). To confirm the precision and accuracy of the elaborated assay, we have compared parameters of the new assay to methods published before, using data indicated for urine and plasma samples (Table 3 and Table 4). As shown in Table 3 and Table 4, the obtained validation data are satisfying. The presented method requires only 50 µL of the sample, while in the previously published methods, much higher volumes of urine or plasma (Table 3 and Table 4) were demanded [30,35,36,38].

The sensitivity of our assay is lower when compared to previously elaborated methods (Table 3 and Table 4); however, we must remember that Crn as a breakdown product of creatine phosphate from muscle and protein metabolism is a typical metabolite present in the human body [21,22]. TFV is a drug administered to HIV and HBV patients. In patients treated with this drug, both compounds are present in quite high concentrations [21,22]. As it was indicated in a previously published method dedicated to the determination of TFV in urine samples, the concentration of the drug was in the range from 0.453 to 43.576 µg mL^−1^ [28]. These data show that our method would be useful for the analysis of those samples and confirm that higher sensitivity would be more required in the case of trace analytes. For this reason, we decided to pay more attention to making it possible to determine both TFV and Crn in one analytical run. That can help us to observe the negative influence of the drug on kidney functions.

For the stability studies, urine and plasma samples were prepared according to the protocols described in Section 4.4. The experiments have proven that TFV and Crn are stable over 180 min in plasma and 240 min in urine at 4 and 37 °C without a noticeable change in the concentration in both cases (Figure 3). RSD for Crn in plasma samples store at 40 and 37 °C was 2.0% and 2.6%, and for TFV, it was 4.7% and 4.5%, respectively. In the case of urine samples kept at 4 and 37 °C, RSD for Crn was 0.4% and 1.0%, and for TFV it was 0.2% and 0.4%, respectively.

The newly elaborated analytical method was used to analyze urine samples collected from CHB patient treated with 123 mg drug dose. The study was mainly focused on the verification of TFV pharmacokinetic. The obtained results clearly indicate that the largest amount of TFV was excreted within 2 to 4 h after taking the drug, and gradually decreased to the eighth hour (Figure 4). Eight hours after the drug was administered, the amount of TFV became constant. It must be highlighted that having a limited number of patients, we cannot provide statistical data to discuss and clearly show a good trend of urinary TFV excretion. However, the validation data clearly indicate that the elaborated method would be useful to carry out this kind of study.

## 4. Materials and Methods

### 4.1. Chemicals

TFV and Crn standards were received from Sigma Aldrich Company (St. Louis, MO, USA). HPLC gradient grade MeCN, sodium hydrogen phosphate heptahydrate, sodium dihydrogen phosphate dihydrate and sodium hydroxide were from J.T. Baker (Deventer, The Netherlands). Perchloric acid was from Merck (Darmstadt, Germany). Deionized water was produced in our laboratory.

### 4.2. Instrumentation

The analyses were performed on 1220 Infinity LC system from Agilent equipped with a binary pump integrated with a two-channel degasser, autosampler, column oven and diode array detector. The samples were injected using the autosampler. Chromatographic separation was achieved on the Zorbax SB C-18 (150 × 4.6 mm, 5 µm) column from Agilent Technologies (Waldbronn, Germany). For instrument control, data acquisition and analysis, OpenLAB software was applied. Water was purified using Milli-QRG system (Millipore, Vienna, Austria). For pH measurement, an HI 221 (Hanna Instruments, Woonsocket, RI, USA) pH meter was used. Precipitated proteins were removed from the sample using Hettich Mikro 200R (Hettich Zentrifugen, Tuttlingen, Germany) centrifuge.

### 4.3. Stock Solutions

Stock solution of 0.3 mg mL^−1^ TFV was prepared in 0.1 mol L^−1^ NaOH [35] and kept at 4 °C for several days without significant changes in the analyte content. A stock solution of Crn 30 µmol mL^−1^ was prepared in deionized water and kept at 4 °C for several days as well. The working solutions were prepared by dilution with deionized water as needed.

### 4.4. Biological Matrices

The urine samples were collected both from healthy people and from CHB patients treated with TFV in the form of Viread 123 mg film-coated tablets. Optimization of analytical conditions were performed on plasma and urine samples collected from healthy volunteers. All samples were stored at −80 °C.

Written informed consent forms were obtained from all volunteers and this study was approved by the Bioethics Committee of the University of Lodz (12/KBBN-UL/I/2015).

#### 4.4.1. Urine Sample Preparation

To reduce the amount of laboratory waste, such as polypropylene tubes or tips, urine samples were prepared directly in reusable HPLC glass vials. For the determination of TFV and Crn to 50 µL of urine, 450 µL of 0.015 mol L^−1^ (pH 7.4) PB was added. Ten microliters of the final analytical solution was injected into the chromatographic column.

#### 4.4.2. Plasma Sample Preparation

The samples were prepared in polypropylene tubes. Fifty microliters of plasma was diluted with 380 µL of 0.015 mol L^−1^ (pH 7.4) PB and spiked with 10 μL of increasing concentrations of TFV. To precipitate plasma proteins, 50 µL of 3 mol L^−1^ perchloric acid was added into the tube. Next, an appropriate amount of deionized water was added to obtain the final volume of 500 µL. The precipitated proteins were removed by centrifugation (15,000× *g*, 10 min, 10 °C). Ten microliters of the supernatant was injected into the chromatographic column.

### 4.5. HPLC Conditions for the Determination of TFV and Crn

#### 4.5.1. Urine Analysis

For chromatographic separation of TFV and Crn in urine, a reversed-phase Zorbax SB C-18 (150 × 4.6mm, 5 µm) column was applied. The analytes were eluted by a mobile phase containing 0.015 mol L^−1^ PB, pH 7.4 and MeCN using gradient as follows: 0–6 min 2–4% MeCN, 6–7 min 4–2% MeCN, 7–8 min 2% MeCN. The flow rate of the mobile phase was 1 mL min^−1^. For the detection of TFV and Crn, two different wavelengths were used—260 and 234 nm, respectively. The total time of the chromatographic analysis was 8 min. For the column reconditioning, 2 min post time was required. The analysis was performed at 25 °C.

#### 4.5.2. Plasma Analysis

The chromatographic separation of TFV and Crn in plasma was obtained in 6 min. The analytes were eluted using isocratic elution with the mobile phase containing 98% of 0.015 mol L^−1^ PB pH 7.4 and 2% MeCN. The flow rate of the mobile phase was 1 mL min^−1^. Similarly to urine analysis, for the detection of TFV and Crn, we used the following wavelength gradient: 0.0–2.5 min 234 nm for Crn and 2.5–6.0 min 260 nm for TFV. The column temperature was 25 °C. Identification of TFV and Crn peaks was based on the comparison of spectrum and retention time of signals with corresponding set of data obtained for authentic compounds.

## 5. Conclusions

For monitoring an adverse reaction of TFV, new analytical tools based on simultaneous separation and quantitation of TFV and Crn in urine and plasma samples have been developed. These assays allow a direct study of correlation between TFV concentration in plasma and urine and an indirect study on the influence of TFV therapy on kidney damage. The assays were fully validated and successfully applied to test urine samples donated by CHB patients treated with TFV administrated as Viread 123 mg.

The most important reason for the application of our assay in laboratories is the possibility to determine TFV and Crn in one analytical run in both urine and plasma samples. The other advantages of the assay, such as (i) the possibility for normalization of the drug concentration against Crn, (ii) a small plasma or urine volume required for analysis, (iii) the total time of the assay for urine analysis less than 10 min, (iv) a reduced amount of laboratory waste, such as tubes/tips/polypropylene vials, and (v) reduction in the amount of toxic organic solvents in the mobile phase, are the added values of the method. The elaborated procedures can be applied for the analysis of samples collected from HIV and HBV/CHB patients and are in the mainstream of new trend of analytical chemistry which is based on green chemistry.

## Figures and Tables

**Table 1:** Calibration data.

Analyte (*n* = 3)	Linear Ranges	Regression Equation	*R* ^2^	RSD (%)		Recovery (%)	
				Min	Max	Min	Max
TFV (µg mL^−1^ urine)	10.0–300.0	*y* = 2.02*x* + 0.47	0.999	1.1	6.1	96.0	108.0
TFV (µg mL^−1^ plasma)	0.5–5.0	*y* = 4.12*x* − 0.06	0.999	2.1	8.4	99.0	100.3
Crn (µmol mL^−1^ urine)	0.1–30.0	*y* = 415.88*x* + 514.96	1.000	0.4	3.1	99.3	111.1
Crn (µmol mL^−1^ plasma)	0.001–0.04	*y* = 1086.71*x* + 24.19	0.999	0.3	4.9	93.6	107.4

**Table 2:** Accuracy and precision.

Analyte	Concentrations	Precision (%)		Accuracy (%)	
		Intra-Day	Inter-Day	Intra-Day	Inter-Day
TFV (µg mL^−1^ urine)	10 40 300	2.3 4.1 1.9	5.8 8.1 4.3	95.9 97.5 100.3	105.7 113.4 96.1
TFV (µg mL^−1^ plasma)	0.5 2 5	4.7 4.1 3.7	5.6 3.6 4.5	91.1 91.5 93.3	93.8 93.5 97.2
Crn (µmol mL^−1^ urine)	0.1 2.5 30	4.2 1.7 3.7	6.8 5.4 6.1	111.4 100.6 99.3	120.1 104.4 108.9
Crn (µmol mL^−1^ plasma)	0.001 0.01 0.04	1.9 0.6 0.5	4.0 5.1 1.4	91.3 100.9 100.7	102.8 99.6 99.3

**Table 3:** Comparison of validation parameters methods for determination of TFV in urine sample.

Parameters	LC-DAD * [30]	LC-MS * [28]	Proposed Method
Sample volume (mL)	0.5	1.0	0.05
Linear range (µg mL^−1^)	1–100	-	10–300
*R* ^2^	0.999	0.999	0.999
LOD (µg mL^−1^)	0.14	0.19	4.0
LOQ (µg mL^−1^)	0.42	0.39	8.0
Intra-day (RSD%)	0.54	6.69	2.8
Inter-day (RSD%)	0.89	9.38	6.1

* The method cannot be applied for the determination of creatinine in urine samples.

**Table 4:** Comparison of validation parameters methods for determination of TFV in plasma sample.

Parameters	LC-DAD * [35]	LC-UV * [36]	Proposed Method
Sample volume (mL)	0.1	1.00	0.05
Linear range (µg mL^−1^)	0.02–10.0	0.01–4.0	0.5–5
*R* ^2^	0.999	-	0.999
LOD (µg mL^−1^)	0.02	0.003	0.15
LOQ (µg mL^−1^)	0.06	0.01	0.5
Intra-day (RSD%)	3.8	5.9	3.7
Inter-day (RSD%)	4.6	8.6	5.6

* The method cannot be applied for the determination of creatinine in plasma samples.
